# 

**Published:** 1877-09

**Authors:** 


### BIBLIOGRAPHICAL.

Micro-Photographs in Histology—Normal and Pathological. By Carl Seiler, M.D., in conjunction with J. Gibbons Hunt, M.D., and Joseph G. Richardson, M.D. Philadelphia, 1877.

This is the ninth number of the first volume of this very estimable publication. We find in it, first, a magnified photograph of a section of lung tissue; and considering the difficulty of accurately defining, in a photograph, a distinct representation of this tissue, we think the work has been creditably performed. Three other plates are embraced in this issue—sections of tubercular lung.

The following pamphlets have been received:

Alcohol as a Food and Medicine. A paper from the Transactions of the International Medical Congress, at Philadelphia, 1876. By Ezra M. Hunt, A.M., M.D., President of the Section of American Medical Association on State Medicine and Public Hygiene, etc.

Report on the Surgical History of Mississippi. By W. W. Hall, M.D. Grenada, Miss., 1877.

Medical Organizations and their Value. An address delivered before the Alumni Association of Jefferson Medical College, March 9, 1877. By Wm. B. Atkinson, M.D.

McDowell Medical College—Sixth Annual Session, 1877.

Address by the President, E. H. Luckett, M.D., of Owensboro, Ky. Read at Madisonville, Ky., May 9, 1877.

Calculi Found in the Bladder—After the Cure of Vesico-Vaginal Fistula. By Henry F. Campbell, M.D., Augusta, Ga. Reprint from Vol. 1 Gynæcological Transactions, 1876.

Pneumatic Self-Replacement of Gravid and Non-Gravid Uterus. By Henry F. Campbell, Augusta, Ga. Reprint from Vol. 1 Gynæcological Transactions, 1876.

History of a Case of Recurring Sarcomatous Tumor of the Orbit in a Child—Extirpated for the third time, and ultimately causing the death of the patient. By Thomas Hay, M.D. Philadelphia. Illustrated. 1877.

Two Cases of Morphœa—With remarks on the disease and its differential diagnosis. By L. Duncan Bulkley, A.M., M.D., Physician to the Skin Department, Demilt Dispensary, New York, etc. G. P. Putnam & Sons. 1877.

Defects of Hearing and other Evils—The Result of Hypertrophied Tonsils, etc. By A. W. Calhoun, M.D., Professor of Diseases of the Eye and Ear and Clinical Ophthalmology and Otology, in the Atlanta Medical College. H. H. Dickson, Atlanta, Ga., 1877.

On the Nomenclature and Classification of Diseases of the Skin. By L. Duncan Bulkley, A.M., M.D., Physician to the Skin Department, Demilt Dispensary, New York, etc. G. P. Putnam & Sons. 1877.

Naphey's Therapeutics.—A new edition (fifth) soon to be published, will consist of two volumes of 600 pages each, viz: 1. Medical Therapeutics; 2. Surgical Therapeutics.

Intra-Uterine Pessaries.—A reply to "A Plea for Woman," in the *Louisville Medical News*, of May 26, 1877. By V. H. Taliaferro, M.D., Professor of Obstetrics and Diseases of Women, in the Atlanta Medical College, etc. 1877.

Inaugural Dissertation on Tetanus, Etc.—For the Degree of Doctor of Medicine and Surgery, in Frederick William University of Berlin, August 15, 1877. By Henry F. Scott, M.D., of Atlanta, Ga.

